# High-Intensity Functional Concurrent Training for Physical Fitness, Body Composition, and Psychological Outcomes in Schoolchildren: Protocol for a Randomized Controlled Trial

**DOI:** 10.3390/sports14070279

**Published:** 2026-07-03

**Authors:** Natalia Durán-López, Carlos Gómez-García, Antonio Ranchal-Sanchez, Valentina Lucena-Jurado, Victoria Moyano-Ortega, Ana Lara-Barahona Ostos, Jose Manuel Jurado-Castro

**Affiliations:** 1 Ministry of Educational Development and Vocational Training, Regional Government of Andalusia, 41092 Sevilla, Spain; nataliaduranlopez@gmail.com; 2 Department of Psychology, Faculty of Educational Sciences and Psychology, University of Cordoba, 14004 Córdoba, Spain; ed1lujuv@uco.es; 3 Osuna University School, Teaching Center Attached to the University of Seville, 41640 Seville, Spain; carlosgg@euosuna.org (C.G.-G.); juradox@gmail.com (J.M.J.-C.); 4 Maimonides Biomedical Research Institute of Cordoba (IMIBIC), Reina Sofia University Hospital, University of Cordoba, 14004 Córdoba, Spain; 5 Department of Nursing, Pharmacology and Physiotherapy, Faculty of Medicine and Nursing, University of Cordoba, 14004 Córdoba, Spain; 6 CIBERobn Physiopathology of Obesity and Nutrition, Centre of Biomedical Research Network, Institute of Health Carlos III, 28029 Madrid, Spain

**Keywords:** high-intensity functional concurrent training, schoolchildren, physical fitness, psychological health

## Abstract

High-intensity functional concurrent training (HIFCT) has emerged as a form of training characterized by constantly varied functional movements adapted to individual fitness levels. Previous studies have reported positive effects on muscular strength, cardiorespiratory fitness, body composition, and psychological well-being; however, evidence regarding HIFCT interventions in school-aged children remains limited. Therefore, the aim of the present study protocol is to evaluate the effects of an 8-week HIFCT programme on muscular strength, cardiorespiratory fitness, body composition, anxiety, stress, and self-esteem in children aged 10–12 years. Physical fitness, anthropometric variables, and psychological outcomes will be assessed before and after the intervention using validated field-based tests and questionnaires. This study may provide novel evidence regarding the feasibility, safety, and potential effects of HIFCT programmes in the school setting. The study protocol was registered at ClinicalTrials.gov (NCT07552844) and approved by the Córdoba Research Ethics Committee (IMIBIC, Córdoba, Spain; protocol code SICEIA-2025-000408).

## 1. Introduction

Childhood and adolescence represent critical periods for physical, psychological, and social development, as well as for the establishment of lifestyle habits that may persist into adulthood [1]. In this context, regular participation in physical activity has been identified as one of the main protective factors against the development of cardiometabolic diseases, psychological disorders, and sedentary behaviors [2]. Nevertheless, a progressive decline in physical activity levels among children and adolescents has been observed over recent decades, with a substantial proportion of young people failing to meet current international health recommendations [3]. This situation has been associated with increasing rates of childhood obesity, deteriorations in physical fitness, and a greater prevalence of emotional problems such as anxiety, stress, and low self-esteem [4].

Current evidence indicates that structured exercise programmes provide significant benefits for both physical and mental health in children and adolescents [5]. In particular, muscular strength and cardiorespiratory fitness are strongly associated with metabolic, functional, and cardiovascular health outcomes [6]. Consequently, international organizations recommend the inclusion of strength and conditioning activities from an early age, provided that they are appropriately supervised and adapted to participants’ developmental characteristics [7,8,9].

Among the exercise modalities currently used to improve health-related fitness, high-intensity functional concurrent training (HIFCT) has emerged as a promising approach. Unlike traditional concurrent training models, which typically separate resistance and aerobic exercise into distinct sessions or training blocks, HIFCT integrates strength and cardiorespiratory stimuli within the same session through functional, multi-joint exercises performed at moderate-to-vigorous intensities [10,11]. This training approach incorporates varied and dynamic activities which may be particularly suitable during childhood by promoting engagement, enjoyment, and sustained participation in physical activity [12,13]. The increasing popularity of HIFCT in both recreational and sports settings has generated growing scientific interest in its potential effects on health and physical performance [14]. Previous studies have reported that HIFCT interventions may improve body composition, muscular strength, and cardiorespiratory fitness, leading to reductions in body fat percentage and increases in lean body mass among adults and physically active children and adolescents [13,14]. These adaptations are particularly relevant during childhood and adolescence, which are considered critical periods for the prevention of metabolic and cardiovascular diseases associated with sedentary behaviors and excess adiposity [5].

Beyond physical health outcomes, regular participation in physical exercise has also been associated with improvements in several psychological outcomes among children and adolescents. Recent systematic reviews indicate that physical activity may contribute to reductions in symptoms of anxiety, stress, and depression, while also improving self-esteem, emotional well-being, perceived physical competence, and health-related quality of life [15,16]. Furthermore, this has been proposed as an attractive exercise modality for children due to its capacity to enhance intrinsic motivation and enjoyment [17,18]. Additionally, the dynamic and cooperative nature of this type of training, together with the variety of tasks and the social component present during the sessions, may promote feelings of group belonging and personal satisfaction [19].

Importantly, the implementation of HIFCT in pediatric populations requires careful adaptation to participants’ developmental characteristics. The age range of 10–12 years is particularly relevant, as this period represents a key stage for the acquisition of fundamental movement skills, motor competence, and physical fitness, as well as for the establishment of long-term physical activity habits [20,21]. Within this developmental context, HIFCT may be a suitable training modality because it combines varied functional movement patterns with strength- and cardiorespiratory-oriented stimuli in a dynamic format that can be individually adapted to children’s abilities and interests. This rationale is consistent with current recommendations from the National Strength and Conditioning Association and the 2014 International Consensus, which support appropriately prescribed, progressive, and supervised youth neuromuscular training programmes in children and adolescents [20,21]. These recommendations are further aligned with pediatric guidance emphasizing technical competency, qualified supervision, and gradual progression in youth training. Importantly, no prospective study of supervised youth training programmes involving strength-oriented exercise has reported growth plate injuries, and supervised training appears to be associated with lower injury rates than many common youth sports [21]. Accordingly, the present intervention was designed as a non-competitive child-oriented HIFCT programme based on bodyweight exercises, light external loads, age-appropriate movement patterns, and individualized progression under professional supervision. Acknowledging the substantial developmental heterogeneity within this age range—particularly regarding motor competence, attention span, and physical capacity—the present protocol incorporates age-stratified training to ensure that programme content is appropriately matched to participants’ developmental stage [21].

Despite the growing popularity of HIFCT and its potential benefits for physical and psychological health, evidence in school-aged children remains limited. Most available studies have been conducted in adult or athletic populations [10], and relatively few randomized controlled trials have examined HIFCT programmes specifically designed for children and adolescents [6,8]. Moreover, substantial heterogeneity exists across studies regarding training structure, exercise selection, intervention duration, supervision procedures, and age-adaptation strategies, which limits the comparability of findings and the ability to draw clear conclusions regarding the feasibility, safety, and effectiveness of child-oriented HIFCT interventions [9]. Furthermore, relatively few studies have simultaneously examined physical fitness, body composition, and psychological outcomes within the same HIFCT intervention in pediatric populations [13,14,15].

The age range of 10–12 years was selected because it represents a developmentally appropriate stage for the implementation of a supervised HIFCT programme involving multi-joint movement patterns, progressive external loading, and competency-based exercise progressions [22]. In addition, recent pediatric resistance training research has specifically targeted schoolchildren aged 10–12 years when designing and evaluating structured resistance-based interventions, supporting the practical and methodological relevance of this age range for supervised strength-oriented programmes [22]. This rationale is further supported by the neuromuscular characteristics of late childhood, a developmental stage in which relevant improvements in force-producing capacity and neuromuscular function have been described [23]. Moreover, strength gains appear to be mediated predominantly by neural rather than hypertrophic mechanisms [20], with reported training-induced adaptations including enhanced motor unit recruitment, higher firing frequency, improved neural drive, and better intra- and intermuscular coordination [24].

Therefore, the aim of the present study protocol is to evaluate the effects of an 8-week HIFCT intervention on muscular strength, cardiorespiratory fitness, body composition, anxiety, stress, and self-esteem in children aged 10–12 years. It is hypothesized that children allocated to the HIFCT intervention will demonstrate greater improvements in muscular strength, cardiorespiratory fitness, body composition, anxiety, stress, and self-esteem than those assigned to the control group.

## 2. Materials and Methods

### 2.1. Study Design

The present study is designed as a randomized controlled trial aimed at evaluating the effects of an 8-week HIFCT programme in school-aged children between 10 and 12 years old. The study protocol follows the SPIRIT [25] (Standard Protocol Items: Recommendations for Interventional Trials) guidelines for clinical trial protocols, including the SPIRIT schedule (Figure S1) and the SPIRIT checklist (Supplementary Table S1). Participants will be randomly assigned to either an experimental group or a control group. Baseline and post-intervention assessments will be performed before (pre-test) and after (post-test) the intervention period. Moreover, the CONSORT (Consolidated Standards of Reporting Trials) statement has been considered in the study design report [26]. Participants allocated to the experimental group will complete a supervised HIFCT programme specifically designed for children aged 10–12 years, with session content, exercise complexity, and training demands adapted to the developmental characteristics and movement competency of this age group.

### 2.2. Study Setting

The study will be conducted in Córdoba (Spain). Participant recruitment and baseline assessments will be performed at a public primary school, while the intervention sessions and physical fitness assessments will be conducted at a specialized HIFCT facility located in Córdoba. The intervention programme will be supervised by professionals specialized in pediatric physical exercise and HIFCT. The center will provide the facilities, equipment, and technical staff required for the intervention at no cost to participants or their families.

### 2.3. Participants

Participants will consist of children enrolled at a public primary school. Recruitment procedures will ensure that none of the participants are engaged in structured extracurricular sports activities before the beginning of the intervention.

Participants will be randomly assigned to one of two study groups: (i) an experimental group, which will participate in an 8-week supervised HIFCT, or (ii) a control group, which will maintain their usual routines without participating in any structured physical exercise programme during the intervention period. To confirm compliance with these criteria, parents or legal guardians will complete a questionnaire regarding their child’s participation in organised physical activities outside school hours, including the weekly frequency and type of activity performed. Families of children assigned to the experimental group will also be instructed to avoid participation in additional structured sports activities during the intervention period.

To be eligible for participation, children must meet the following inclusion criteria: (i) be between 10 and 12 years old; (ii) be enrolled at a public primary school; (iii) not participate in extracurricular sports activities before the beginning of the intervention; (iv) be available to attend two weekly training sessions during the 8-week intervention period in the case of the experimental group; (v) present no medical, psychological, or behavioral contraindications that may limit participation in physical exercise; and (vi) provide written informed consent signed by parents or legal guardians.

Exclusion criteria will include: (i) musculoskeletal injuries, chronic diseases, or medical conditions limiting participation in physical exercise; (ii) formally diagnosed psychological, behavioral, or neurodevelopmental disorders that may interfere with safe participation in the intervention or with the completion of the outcome assessments, as reported by parents or legal guardians during the screening process; (iii) inability to attend the intervention sessions regularly; (iv) participation in structured extracurricular sports activities during the intervention period; and (v) absence of written informed consent from parents or legal guardians. Before participation, families will receive detailed written and verbal information regarding the study aims, procedures, benefits, and possible risks. Written informed consent signed by parents or legal guardians will be required before enrollment. Contact information will also be provided to resolve any questions related to the study procedures.

### 2.4. Randomization and Allocation Concealment

Following eligibility assessment and baseline evaluations, participants will be randomly assigned to either the experimental group or the control group in a 1:1 ratio. Randomization will be performed using a centralized computer-based randomization system (Sealed Envelope™, London, UK; https://www.sealedenvelope.com). A block randomization procedure with randomly varying block sizes will be used to maintain balance between groups while preserving allocation unpredictability. In view of the expected sample size, stratified randomization will be performed by sex and age, ensuring balanced allocation of both variables across the experimental and control groups.

The allocation sequence will be generated by a researcher not involved in participant recruitment, baseline assessments, intervention delivery, or outcome evaluation. The investigators responsible for recruitment and baseline assessments will remain blinded to the allocation sequence and block sizes until participants have been enrolled and baseline measurements have been completed. Once assigned, group allocation will not be modified throughout the intervention period.

### 2.5. Blinding

Due to the nature of the exercise intervention, participants, families, and trainers cannot be blinded to group allocation. However, outcome assessors will be blinded to group allocation during baseline and post-intervention assessments. Participants and families will be instructed not to disclose their group allocation to the assessors during the post-intervention evaluations. Data analysis will be performed using coded group labels whenever feasible to reduce analytical bias.

### 2.6. Control of Contamination and Lifestyle-Related Factors

Because participants will be recruited from the same school, families in both groups will be asked to maintain their children’s usual routines during the intervention period. Control-group participants will be asked not to start any new structured exercise programme during the study. Likewise, families in the experimental group will be asked to avoid additional structured training outside the intervention.

Although diet, sleep, spontaneous physical activity, and screen time will not be objectively monitored, families will be asked to avoid intentional changes in these habits during the intervention period. Any relevant change in extracurricular physical activity, diet, sleep routines, or screen time reported by families will be recorded descriptively and considered when interpreting the results.

### 2.7. Ethical and Legal Aspects

The present study protocol was registered at ClinicalTrials.gov under the identifier NCT07552844 and approved by the Córdoba Research Ethics Committee (IMIBIC, Córdoba, Spain) under protocol code SICEIA-2025-000408. The study will be conducted in accordance with the ethical principles of the Declaration of Helsinki (World Medical Association, 2013), [27] the principles of Good Clinical Practice (ICH-GCP), [28] and the current Spanish and European legislation governing biomedical research involving human participants, including Law 14/2007 of 3 July on Biomedical Research [29], Royal Decree 1090/2015 regulating clinical trials with medicinal products (if applicable) [30], Regulation (EU) 2016/679 of the European Parliament and of the Council (General Data Protection Regulation) [31], and Organic Law 3/2018 of 5 December on Personal Data Protection and Guarantee of Digital Rights [32].

Before participation, parents or legal guardians will receive detailed written and verbal information regarding the study aims, procedures, potential benefits, and possible risks associated with participation. Written informed consent signed by parents or legal guardians will be required prior to enrollment. Participation in the study will be completely voluntary, and participants may withdraw from the study at any time without consequences.

Participant confidentiality and anonymity will be strictly guaranteed throughout the entire research process. All collected data will be anonymized and codified using numerical identification codes to prevent participant identification. Only the research team will have access to the study data, which will be securely stored and protected from unauthorized access in accordance with current data protection regulations. Coaches, teachers, and non-research staff will not have access to individual participant information or study results.

In accordance with Spanish data protection legislation, parents or legal guardians of participants under 14 years of age may exercise the rights of access, rectification, erasure, restriction of processing, data portability, and opposition concerning the personal data of the participants. Any request related to these rights may be addressed directly to the research team.

All intervention sessions and assessment procedures will be supervised by qualified professionals specialized in pediatric physical exercise and HIFCT in order to ensure participant safety and minimize the risk of injury during exercise practice. In addition, the study will follow quality assurance and Good Clinical Practice procedures throughout all phases of the research process. Before the start of the intervention, all professionals involved in programme delivery will be familiarized with the study protocol, session structure, exercise progressions, and safety procedures to ensure standardized implementation.

### 2.8. Procedures

#### 2.8.1. Training for Researchers

Before the beginning of the study, all researchers and exercise professionals involved in the intervention and assessment procedures will participate in a standardized training session. The training will focus on the correct administration of physical fitness tests, anthropometric measurements, and psychological questionnaires in order to ensure consistency and reliability across all assessments. Special attention will be given to the standardization of testing procedures, participant safety, exercise supervision, and data collection protocols. In addition, the research team will review the correct use of all assessment equipment and the procedures established for the intervention sessions.

#### 2.8.2. Intervention

The intervention programme will last 8 weeks and will consist of two weekly sessions of approximately 50 min each. Participants assigned to the experimental group will complete a supervised HIFCT programme specifically adapted to the age and physical capacities of school-aged children. Training sessions will be conducted at Triple XXX CrossFit facilities (Córdoba, Spain) and supervised by professionals specialized in pediatric physical exercise and HIFCT to ensure participant safety, correct exercise execution, and injury prevention. Each session will include HIFCT exercises combining strength, coordination, balance, and cardiorespiratory conditioning using age-adapted equipment such as plyometric boxes, rowing and ski ergometers, skipping ropes, medicine balls, and body-weight exercises. The intervention program will be structured into four main sections: (i) warm-up; (ii) strength training; (iii) metabolic conditioning (METCON); and (iv) cool-down activities (Table 1) [33]. Sessions will incorporate greater movement complexity, longer work intervals, progressive introduction of light external loads (medicine balls, resistance bands) from week 3 onwards, and a higher training density consistent with their greater attentional capacity and motor maturity. This differentiation is consistent with the Youth Physical Development model and current long-term athletic development frameworks, which recommend that training content be tailored to developmental stage rather than chronological age alone [20,21].

The warm-up phase (15 min) will include mobility exercises and dynamic games aimed at increasing body temperature and preparing participants for exercise. The strength block (15 min) will focus on movement technique and age-adapted strength exercises. The METCON component (10 min) will consist of functional circuits performed at moderate-to-vigorous intensity, combining aerobic and muscular endurance exercises. Finally, the cool-down phase (5–10 min) will include games, mobility exercises, and recovery activities [20] (Table 2). Exercise sessions will be performed at a target moderate-to-vigorous intensity. Session density will be standardized through the fixed structure described above. Work-to-rest ratios will vary according to the objectives and characteristics of each session, ranging from continuous AMRAP formats to circuit-based activities incorporating recovery periods of approximately 30–60 s between rounds.

Internal training load and perceived exercise intensity will be monitored after each session using the Pictorial Children’s Effort Rating Table (PCERT) [34], allowing training demands to be adjusted when necessary. Training load will be progressively increased throughout the intervention by manipulating exercise volume (number of repetitions and rounds), movement complexity, external resistance when appropriate, and work-to-rest ratios, while maintaining age-appropriate exercise demands and correct movement execution. The progression strategy implemented across the 8-week intervention is summarized in Table 1. Exercises will be individually adapted according to participants’ motor competence, physical fitness level, technical proficiency, and developmental characteristics. Coaches will apply a three-stage progression model for teaching movement patterns: (i) movement exploration and body awareness; (ii) technique instruction through verbal cues, visual demonstration, and manual guidance; and (iii) technique consolidation under supervised low-complexity practice. Progression to higher-complexity or loaded variations will only occur once correct execution of the fundamental pattern has been confirmed by the supervising coach. Adaptations may include modifications to exercise complexity, range of motion, movement execution, training volume, or recovery periods to ensure safe and appropriate participation. Attendance will be recorded at every training session. Participants attending at least 80% of the scheduled sessions will be considered adherent to the intervention protocol. Any missed sessions and the reasons for absence will also be documented throughout the study period.

Exercise selection, movement complexity, external loading, METCON duration, and work density will be adapted according to age, motor competence, and technical proficiency. Adaptations may include modifications to exercise complexity, range of motion, movement execution, training volume, or recovery periods to ensure safe and appropriate participation.

The volume and intensity of each session will be designed in accordance with current recommendations for youth resistance and neuromuscular training in untrained school-aged children. The NSCA recommends that beginners start with 1 set of 10–15 repetitions per exercise, progressing gradually to 1–3 sets of 6–15 repetitions as technical proficiency and tolerance develop [20]. Accordingly, the strength component of the intervention will generally be prescribed within a range of 2–3 sets per exercise across the programme, with exercise complexity, external loading, and selected training volumes progressed according to technical competency, session objectives, and individual tolerance. In parallel, the volume and density of jumping tasks and METCON activities will be prescribed conservatively and progressively adjusted across the intervention. During the initial sessions, lower-complexity jumping tasks, moderate jump density, and shorter METCON formats will be prioritised, whereas more demanding jumping variations and longer conditioning blocks will be introduced progressively in the later sessions when the supervising coach confirms adequate movement control, landing mechanics, and tolerance to training load. Thus, higher-impact variations such as tuck jumps or jump lunges will be delayed, simplified, or replaced whenever they are considered inappropriate for the participant’s technical competency or fatigue level. Internal load will be monitored after each session using the PCERT [34], and the supervising coach will be allowed to reduce exercise volume, movement complexity, work density, or introduce brief intra-session pauses whenever a session is considered excessively demanding for the participant’s competency or fatigue level.

Exercise progression will follow a competency-based approach, whereby progression to more technically demanding or externally loaded exercises will only occur after participants demonstrate adequate movement quality and postural control during prerequisite tasks. Initial training will emphasize bodyweight exercises, unloaded movement patterns, elastic-band exercises, and low-load medicine-ball activities. External resistance and exercise complexity will be progressively increased according to individual technical competency, as judged by the supervising coach using predefined movement-quality criteria. Jumping tasks and metabolic conditioning exercises will likewise be adapted according to movement competency, ensuring that exercise selection remains appropriate for each participant throughout the intervention. This progression model was designed according to current recommendations for youth resistance training and long-term athletic development [20,21].

An adverse event will be defined as any injury, pain, discomfort, illness, or other undesirable occurrence arising during participation in the intervention sessions. Participation may be temporarily or permanently suspended if a participant experiences a medical condition or adverse event that compromises safe participation, as determined by the research team and, when appropriate, by the participant’s healthcare provider. All training sessions will be conducted at facilities equipped with first-aid resources and supervised by qualified personnel trained to follow the standard emergency procedures established by the facility [20,21]. Participants assigned to the control group will not participate in the intervention programme and will maintain their usual daily routines throughout the study period.

Attendance will be recorded at every training session. Participants attending at least 80% of the scheduled sessions will be considered adherent to the intervention protocol. Any missed sessions and the reasons for absence will also be documented throughout the study period.

### 2.9. Outcomes

All outcome variables will be assessed at baseline (pre-test) and immediately after the 8-week intervention period (post-test). The study will evaluate physical fitness, body composition, and psychological outcomes in children aged 10–12 years using validated field-based tests and questionnaires (Figure S2). All baseline and post-intervention assessments will be completed during a single evaluation session lasting approximately 60–90 min. To minimize fatigue, physical fitness tests will be administered in a standardized order with adequate recovery periods between assessments according to established testing protocols. Participants will receive standardized instructions and verbal encouragement throughout the testing session. Familiarization trials will be provided when required by the testing protocol to ensure adequate understanding of the procedures and to promote valid test performance. Before the strength assessments, all participants will perform a standardized warm-up protocol consisting of: (i) a general warm-up including light running, skipping, heel-to-glute movements, arm circles, wrist and ankle rotations, lunges, and changes in direction; (ii) a specific warm-up for jump including controlled squats, low-intensity jumps, and lunges with trunk rotation; (iii) a specific warm-up for handgrip dynamometry involving squeezing a rubber ball and wrist flexion-extension exercises; and (iv) a specific warm-up for the medicine ball throw including shoulder rotations, light medicine ball throws, and core activation exercises.

#### 2.9.1. Primary Outcome

##### VO_2_max (Course-Navette Test)

Cardiorespiratory fitness will be assessed using the 20-m Course-Navette test, a validated field-based test widely used to estimate maximal oxygen uptake (VO_2_max) in children and adolescents [35]. Participants will run continuously between two lines separated by 20 m at progressively increasing speeds, starting at 8.5 km·h^−1^ and increasing by 0.5 km·h^−1^ every minute. The test will be terminated when participants fail to reach the line in time on two consecutive occasions or voluntarily stop due to fatigue. VO_2_max will subsequently be estimated from the final stage attained using the equation developed by Léger et al. [35].

#### 2.9.2. Secondary Outcomes

##### Lower-Limb Explosive Strength (Countermovement Jump Test)

Lower-limb explosive strength will be assessed using the countermovement jump (CMJ) test described by Bosco et al. [36]. Participants will begin from an upright standing position with their hands placed on the waist and will perform a maximal vertical jump following a rapid downward movement until approximately 90° knee flexion. Three attempts will be performed, and the highest jump height will be recorded. Jump performance will be measured using the ADR Jumping system (ADR, Toledo, Spain).

##### Maximal Isometric Handgrip Strength (Handgrip Dynamometry)

Maximal isometric handgrip strength will be assessed using a digital dynamometer (Takei 5401, Takei Scientific Instruments Co., Ltd., Niigata, Japan) adjusted to the participant’s hand size. Participants will perform the test with the arm extended parallel to the trunk while exerting maximal force. Following one familiarization attempt and one minute of recovery, two attempts will be performed alternately with both hands. The best value obtained for each hand will be recorded [37].

##### Horizontal Lower-Body Explosive Strength (Standing Long Jump)

Horizontal explosive strength will be assessed using the standing long jump test. Participants will perform a maximal horizontal jump using both feet simultaneously without a preparatory step. The longest distance achieved will be measured using a measuring tape from the starting line to the nearest heel at landing. Two attempts will be performed, and the best result will be recorded [38].

##### Upper-Body Explosive Strength (Medicine Ball Throw)

Upper-body explosive strength will be assessed using a 2-kg medicine ball throw test. Participants will stand upright holding the medicine ball with both hands above the head and will throw it forward as far as possible without crossing the designated line. Two attempts will be performed, and the best distance achieved will be recorded [39].

##### Core Muscular Endurance (Plank Test)

Core muscular endurance will be assessed using the plank test. Participants will maintain a prone plank position supported by forearms and toes while maintaining a neutral spine and body alignment. The test will end when participants fail to maintain the correct position after verbal correction. The maximum time maintained in the correct position will be recorded [40].

##### Muscular Endurance and Functional Capacity (Burpees Test)

Muscular endurance and functional capacity will be evaluated using a burpee test performed for one minute. Participants will complete as many correctly executed burpees as possible during the established time period. The total number of repetitions completed will be recorded [41].

##### Cardiovascular Recovery Capacity (3-min Step Test)

Cardiovascular recovery capacity will be assessed using a 3-min step test performed on a 30-cm step. Participants will step continuously at a standardized cadence throughout the test duration. Heart rate will be recorded immediately after exercise and following 1 min of passive recovery in order to evaluate post-exercise cardiovascular recovery [42].

##### Age and Sex

Participants’ age and sex will be recorded before baseline assessments.

##### Body Weight, Height, and Body Mass Index (BMI)

Body weight and height will be assessed according to the ALPHA-Fitness battery procedures [43]. Body weight will be measured using an electronic scale (Tanita RD-545; Tanita Corporation, Tokyo, Japan), and height will be measured using a portable stadiometer (SECA 213; Seca GmbH & Co. KG, Hamburg, Germany). Body mass index (BMI) will subsequently be calculated as body weight in kilograms divided by height in meters squared (kg/m^2^).

##### Body Fat Percentage

Body fat percentage will be assessed using bioelectrical impedance analysis according to the International Society for the Advancement of Kinanthropometry (ISAK) guidelines. Participants will be assessed barefoot and wearing light clothing following standardized anthropometric procedures [44].

Psychological assessments will be administered on the same day as the physical evaluations at baseline and post-intervention. Participants will complete the questionnaires in a quiet and controlled environment under researcher supervision.

##### Anxiety (State–Trait Anxiety Inventory for Children; STAIC)

Anxiety will be assessed using the State–Trait Anxiety Inventory for Children (STAIC), developed by Spielberger et al. [45] and adapted into Spanish by Gualberto et al. [46]. The questionnaire evaluates both state anxiety and trait anxiety through 40 items divided into two subscales. Previous studies have shown adequate internal consistency for the instrument, with a Cronbach’s alpha coefficient of approximately 0.70.

##### Daily Stress (Children’s Daily Stress Inventory; IECI)

Stress levels will be assessed using the Inventario de Estrés Cotidiano Infantil (IECI; Children’s Daily Stress Inventory) developed by Trianes et al. [47]. The IECI is composed of 22 dichotomous items (yes/no response format) designed to evaluate daily stress in the domains of health, school, and family. The instrument showed adequate internal consistency, with a Cronbach’s alpha coefficient of 0.81 for the total scale, while subscale coefficients ranged from 0.62 to 0.68.

##### Self-Esteem (Rosenberg Self-Esteem Scale)

Self-esteem will be assessed using the Rosenberg Self-Esteem Scale [48], adapted into Spanish by Vázquez and Rodríguez [49]. The scale includes 10 items answered using a 4-point Likert scale and showed adequate internal consistency (Cronbach ’s α = 0.87).

Although all psychological instruments selected for this study have been previously validated for use in pediatric populations, some measures may show lower sensitivity in younger children. Therefore, the results of the psychological assessments will be interpreted taking participants’ age and developmental characteristics into consideration [50].

##### Perceived Exertion (Pictorial Children’s Effort Rating Table; PCERT)

Perceived exertion during exercise will be assessed using the Pictorial Children’s Effort Rating Table (PCERT) [34]. Participants will identify the image corresponding to their perceived level of effort after exercise sessions. The instrument showed adequate reliability for assessing perceived exertion in children (Cronbach’s α => 0.70).

### 2.10. Sample Size and Power

Sample size estimation was performed using G*Power software version 3.1 [51] to ensure adequate statistical power to detect significant differences between the experimental and control groups in one of the primary outcomes of the study, maximal oxygen uptake (VO_2_max) estimated from the Course-Navette test. The calculation was based on two-tailed independent samples t-test using data reported by Alves et al. [52], who conducted a concurrent training intervention in children of similar age and intervention duration. In that study, the intervention group showed a mean increase of 1.7 mL·kg^−1^·min^−1^ in VO_2_max, whereas the control group showed a mean increase of 0.2 mL·kg^−1^·min^−1^. The pooled standard deviation of the change scores was calculated from the reported standard deviations of the intervention and control groups, 1.9 and 1.6, respectively, resulting in a pooled SD of approximately 1.76. Based on these values, the estimated standardized effect size was Cohen’s d = 0.86. Assuming an alpha level of 0.05, a statistical power of 0.80, and an allocation ratio of 1:1, the required sample size was estimated at 46 participants, with 23 participants per group. Considering a potential dropout rate of approximately 10–15%, the target recruitment will be increased to approximately 52–54 participants to maintain adequate statistical power throughout the study period.

### 2.11. Statistical Analysis

Statistical analyses will be performed using IBM SPSS Statistics version 26.0 (IBM Corp., Armonk, NY, USA) or equivalent statistical software. Descriptive statistics will be calculated for all study variables and presented as mean ± standard deviation or median and interquartile range for continuous variables, and as frequencies and percentages for categorical variables. Data distribution will be checked using graphical methods and, when appropriate, the Shapiro–Wilk test. To account for the repeated-measures design, intervention effects will be analyzed using linear mixed-effects models. Separate models will be performed for each outcome, including group, time, and the group × time interaction as fixed effects, and participant ID as a random effect. Models will be adjusted for age, sex, and growth-related variables such as baseline height, body mass, and body mass index. Effect sizes will be calculated using partial eta squared (η^2^p) and interpreted as small (0.01), medium (0.06), or large (0.14) effects. In addition, Hedges’ g effect sizes will be calculated to estimate the magnitude of within-group changes and between-group differences in pre–post changes [53]. All effect-size estimates will be reported with 95% confidence intervals whenever appropriate.

Analyses will primarily follow the intention-to-treat principle. Linear mixed-effects models will be used because they allow the inclusion of all randomized participants with available observations and can accommodate incomplete outcome data under the missing-at-random assumption. Therefore, no imputation procedures are planned unless the amount or pattern of missing data suggests otherwise. A per-protocol analysis will also be conducted as a sensitivity analysis including participants who attend at least 80% of the intervention sessions.

Relevant changes in lifestyle-related factors during the intervention period, including the initiation of new structured physical activity outside the study, substantial changes in diet, sleep routines, or screen time, will be recorded when reported by families and considered when interpreting the results. Sensitivity analyses will be performed when appropriate and feasible.

Considering the number of physical fitness, body composition, and psychological outcomes included in the study, the possibility of inflated type I error due to multiple comparisons cannot be excluded and will be considered when interpreting the results. This issue will also be acknowledged as a study limitation. Statistical significance will be established at *p* < 0.05 for all analyses.

## 3. Discussion

The present study protocol describes an 8-week HIFCT intervention designed to evaluate its effects on physical fitness, body composition, and psychological outcomes in school-aged children. Despite the growing scientific interest in HIFCT in pediatric populations, the available evidence remains limited and, in some cases, inconsistent [10,11,12]. Therefore, the present study seeks to contribute to the current literature by examining the feasibility, safety, and potential effects of a supervised and developmentally appropriate HIFCT programme in children aged 10–12 years.

Current evidence supports the role of regular physical activity in promoting physical and mental health during childhood and adolescence [2,5]. Previous studies have shown that structured exercise programmes can improve muscular strength, cardiorespiratory fitness, and body composition in youth populations [6]. In particular, resistance and concurrent training interventions have demonstrated positive effects on several components of physical fitness, although the magnitude of these adaptations appears to depend on factors such as training volume, intensity, participant characteristics, and programme design [9]. Within this context, HIFCT has emerged as a training modality that integrates functional multi-joint movements with strength- and cardiorespiratory-oriented stimuli within the same session [7,8].

Although several studies have reported favorable effects of HIFCT on physical performance and body composition [13], the available evidence remains heterogeneous. Differences in intervention duration, exercise intensity, participant characteristics, and methodological approaches make direct comparisons between studies difficult [10,11]. Furthermore, body composition outcomes during childhood may be influenced by factors beyond the intervention itself, including biological growth, dietary habits, sleep patterns, and habitual physical activity levels [5]. Consequently, additional well-designed studies are needed to better understand the potential effects of HIFCT in pediatric populations.

Another important aspect of the present protocol is the inclusion of psychological outcomes. Previous research suggests that physical activity may contribute to improvements in self-esteem and emotional well-being, as well as reductions in anxiety and stress symptoms among children and adolescents [15,16]. However, psychological responses to exercise are multifactorial and may depend on factors such as enjoyment, social support, perceived competence, motivational climate, and baseline psychological status [17]. Although some studies have reported positive psychological responses following HIFCT participation [19], findings remain inconclusive, highlighting the need for further investigation in this area.

The safety of HIFCT in children also warrants consideration. Current position statements indicate that resistance and functional training can be safely implemented in pediatric populations when programmes are appropriately supervised, individualized, and progressively prescribed [6,7]. Accordingly, the intervention proposed in the present study incorporates age-appropriate exercise selection, individualized adaptations, professional supervision, progressive overload, and systematic monitoring of adherence, exercise intensity, and adverse events throughout the intervention period.

One of the main strengths of this protocol is the comprehensive assessment of both physical and psychological outcomes within a single intervention. In addition, the programme has been specifically designed according to current recommendations for youth exercise training and includes detailed procedures regarding progression, monitoring, adherence, and participant safety. These characteristics may contribute to a better understanding of the feasibility and potential application of HIFCT in school-aged children.

Several limitations should also be acknowledged. First, the recruitment from a single educational center may limit the generalizability of the findings. Furthermore, the intervention will be conducted in a specialized HIFCT facility rather than within the school environment. Although this setting allows standardized implementation, professional supervision, and access to appropriate equipment, it may limit the ecological validity, scalability, and direct transferability of the findings to typical school-based physical activity programmes. Second, although the intervention duration was selected based on previous studies [15,16], it may be insufficient to detect stable adaptations in some body composition and psychological outcomes. Third, the use of a passive control group, while facilitating implementation under real-world school conditions, may limit the ability to distinguish the specific effects of the HIFCT intervention from the potential influence of increased supervision, social interaction, motivation, or participation in structured physical activity. Moreover, due to the nature of the intervention, participant and trainer blinding is not feasible, which may increase the risk of performance bias. In addition, although age- and growth-related variables will be considered in the analyses, the influence of normal growth and maturation during childhood cannot be completely excluded. Finally, the inclusion of multiple physical fitness, body composition, and psychological outcomes may increase the risk of type I error due to multiple statistical comparisons. Although this comprehensive assessment represents one of the strengths of the study, findings, particularly those related to secondary outcomes, will be interpreted with appropriate caution. Overall, the present study may provide valuable information regarding the feasibility, safety, and potential effects of HIFCT in school-aged children. The findings may contribute to the design of future randomized controlled trials and help inform evidence-based exercise recommendations for pediatric populations.

## 4. Conclusions

This study protocol describes an 8-week HIFCT intervention designed to evaluate its feasibility, safety, and potential effects on muscular strength, cardiorespiratory fitness, body composition, and psychological outcomes in school-aged children. Considering the increasing interest in HIFCT modalities in pediatric populations, this study may provide novel evidence regarding the implementation of developmentally appropriate HIFCT programme in children aged 10–12 years.

The integration of both physical and psychological outcomes represents one of the main strengths of the present protocol and may contribute to a broader understanding of the potential effects associated with HIFCT during childhood. Furthermore, the structured and supervised nature of the intervention may provide valuable information regarding the feasibility and safety of implementing age-appropriate HIFCT strategies in school and community settings.

The findings of this study may contribute to the growing evidence base on HIFCT in pediatric populations and help inform the design of future randomized controlled trials. Nevertheless, further large-scale and long-term studies will be required to confirm the findings and establish evidence-based recommendations regarding the use of HIFCT in children and adolescents.

## Figures and Tables

**Table 1:** Structure and progression of the High-Intensity Functional Concurrent Training intervention during the 8-week study period.

Weeks	Section	General Description	Intensity
1–2	Warm-up	Dynamic stretching, skipping, coordination games	Moderate
1–2	Strength	Basic bodyweight movement patterns and introductory multi-joint exercises, with emphasis on technique instruction, movement control, and familiarisation with loaded exercise patterns when appropriate	Moderate
1–2	METCON	Short circuit-based or AMRAP tasks combining basic locomotor, coordination, jumping, and bodyweight conditioning activities	Moderate
3–4	Warm-up	Dynamic stretching, skipping, coordination games, and mobility drills.	Moderate
3–4	Strength	Introduction of light external loads and more complex multi-joint movement patterns with progressive technical instruction.	Moderate–vigorous
3–4	METCON	Progression to longer or denser conditioning circuits and AMRAP tasks with greater movement complexity and moderate use of jumping activities.	Moderate–vigorous
5–8	Warm-up	Dynamic stretching, skipping, coordination games, and mobility drills.	Moderate
5–8	Strength	Progressive external loading and greater movement complexity in multi-joint exercises.	Moderate–vigorous
5–8	METCON	Longer and more demanding AMRAPs or conditioning circuits, with greater jump complexity, exercise density, and overall conditioning demands when competency criteria are met.	Moderate–vigorous

Note: The intervention programme consisted of two weekly sessions specifically adapted to the age and physical capacities of the participants. Training load and exercise complexity progressively increased throughout the intervention period while maintaining participant safety and appropriate supervision. AMRAP = As Many Rounds As Possible; METCON = metabolic conditioning. All sessions were supervised by qualified professionals specialized in pediatric physical exercise and functional training.

**Table 2:** High-Intensity Functional Concurrent Training intervention programme.

Session	Strength Component (Volume)	METCON Component	Intensity	Recovery
S1	Air squat (2 × 10). Focus on squat mechanics, trunk control, and movement familiarisation.	3 rounds: 10 squats, 10 lateral jumps, 20 m bear crawl	Moderate	1 min between rounds
S2	Incline or knee push-ups (2 × (8–10)). Progress range of motion as appropriate.	AMRAP 6 min: 5 push-ups, 10 lateral jumps, 5 spider walks	Moderate	Continuous
S3	Deadlift pattern with wooden bar/light training bar (2 × (8–10)). Focus on hip-hinge mechanics, trunk control, and movement coordination.	3 rounds: 10 deadlift-pattern reps, 20 jumps, 20 m run.	Moderate	30 s between rounds
S4	Elastic-band row or medicine ball row/pull variation ((2–3) × 10, 1–2 kg if medicine ball is used).	AMRAP 6 min: 8 rows, 10 jumps, 20 s plank	Moderate	Continuous
S5	Shoulder press or medicine ball chest press (2 × (8–10), 1–2 kg), with more complex variations only if technical criteria are met.	3 rounds: 10 presses, 15 jumps, 10 m run	Moderate	Continuous
S6	Lunges (2 × 8/leg). Progress depth and load if appropriate.	4 rounds: 8 lunges, 10 jumps, 10 m crawl.	Moderate	Continuous
S7	Jump squat (2 × 8).	AMRAP 8 min: 5 jump squats, 10 jumps, 10 m run.	Moderate–high	Continuous
S8	Medicine ball throw or push-press variation (2 × (8–10), 1–2 kg) if technical criteria are met.	4 rounds: 8 throws/presses, 12 lateral jumps, 20 m run.	Moderate–high	Continuous
S9	Kettlebell swing (2 × 8, 4–6 kg) only if hip-hinge competency and trunk control are consistently demonstrated, otherwise deadlift-pattern drill with wooden bar or light training bar.	AMRAP 8 min: deadlift-pattern reps or medicine ball slams, 10 squats, 15 m run.	Moderate–high	Continuous
S10	Goblet squat (2 × (8–10), 4–5 kg).	3 rounds: 10 squats, 15 jumps, 10 m duck walk.	Moderate–high	1 min between rounds
S11	Incline or standard push-ups (2 × (8–10)).	AMRAP 10 min: 5 push-ups, 10 m bear crawl, 10 tuck jumps.	Moderate–high	Continuous
S12	Loaded deadlift with wooden bar/light training bar (2 × (8–10)). Load progressed conservatively according to technique.	3 rounds: 10 deadlift-pattern reps, 15 m zigzag run, 8 squat thrusts or burpees if technically appropriate.	Moderate–high	30 s between rounds
S13	Dumbbell shoulder press (2 × 8, 2–3 kg total load). Progress load based on technique.	4 rounds: 8 lunges, 10 box step-ups/jumps, 10 m crawl.	Moderate–high	Continuous
S14	Jump squat (2 × 8).	AMRAP 8 min: 5 jump squats, 10 jumps, 10 m crab walk.	Moderate–high	Continuous
S15	Goblet squat (4–6 kg) or deadlift with light training bar (3 × (6–10)), depending on technical competency and planned progression.	3 rounds: 10 deadlift-pattern reps, 8 jumps, 10 m zigzag run	Moderate–high	30 s between rounds
S16	Jump lunges (2 × 8/leg) only if landing mechanics and unilateral control are adequate; otherwise alternating reverse lunges.	4 rounds: 8 jump lunges, 10 lateral jumps, 10 m bear crawl.	Moderate–high	Continuous

Note: AMRAP = as many rounds as possible; METCON = metabolic conditioning; Exercises involving external loads (e.g., kettlebell swing, goblet squat, and medicine ball variations) will only be introduced following confirmation of prerequisite movement competency by the supervising coach.

## Data Availability

The data generated and analyzed during the current study will be available from the corresponding author upon reasonable request. Data will not be publicly available due to ethical and privacy restrictions involving minors.

## Supplementary Materials

The following supporting information can be downloaded at: https://www.mdpi.com/article/10.3390/sports14070279/s1, Table S1: SPIRIT 2025 checklist of items to address in a randomized trial protocol [25]; Figure S1: SPIRIT schedule of enrollment, interventions, and assessments; Figure S2: outcomes.

## Abbreviations

The following abbreviations are used in this manuscript:

ANOVA	Analysis of Variance
BMI	Body Mass Index
CMJ	Countermovement Jump
CONSORT	Consolidated Standards of Reporting Trials
GC	Control Group
GE	Experimental Group
HIFCT	High-Intensity Functional Concurrent Training
IECI	Inventario de Estrés Cotidiano Infantil
ISAK	International Society for the Advancement of Kinanthropometry
METCON	Metabolic Conditioning
RCT	Randomized Controlled Trial
SPIRIT	Standard Protocol Items: Recommendations for Interventional Trials
STAIC	State–Trait Anxiety Inventory for Children
VO_2_max	Maximal Oxygen Uptake

## References

[1] Sawyer S.M., Azzopardi P.S., Wickremarathne D., Patton G.C. (2018). The age of adolescence. Lancet Child Adolesc. Health.

[2] World Health Organization (2022). Global Status Report on Physical Activity 2022.

[3] Guthold R., Stevens G.A., Riley L.M., Bull F.C. (2020). Global trends in insufficient physical activity among adolescents. Lancet Child Adolesc. Health.

[4] Rodríguez-Ayllon M., Cadenas-Sánchez C., Estévez-López F., Muñoz N.E., Mora-Gonzalez J., Migueles J.H., Molina-García P., Henriksson H., Mena-Molina A., Martínez-Vizcaíno V. (2019). Role of physical activity and sedentary behavior in the mental health of preschoolers, children and adolescents: A systematic review and meta-analysis. Sports Med..

[5] Ortega F.B., Ruiz J.R., Castillo M.J., Sjöström M. (2020). Physical fitness in childhood and adolescence: A powerful marker of health. Int. J. Obes..

[6] Granacher U., Lesinski M., Büsch D., Muehlbauer T., Prieske O., Puta C., Gollhofer A., Behm D.G. (2016). Effects of resistance training in youth athletes on muscular fitness and athletic performance: A conceptual model for long-term athlete development. Front. Physiol..

[7] Smith J.J., Eather N., Morgan P.J., Plotnikoff R.C., Faigenbaum A.D., Lubans D.R. (2014). The health benefits of muscular fitness for children and adolescents: A systematic review and meta-analysis. Sports Med..

[8] Gäbler M., Prieske O., Hortobágyi T., Granacher U. (2018). The effects of concurrent strength and endurance training on physical fitness and athletic performance in youth: A systematic review and meta-analysis. Front. Physiol..

[9] Cui F., Sun Z., Ma M., Dong A., Xu C., Zhu J., Ma G. (2026). The effects of concurrent training on physical fitness in children and adolescents: A systematic review and meta-analysis. Front. Pediatr..

[10] Claudino J.G., Gabbett T.J., Bourgeois F., Souza H.d.S., Miranda R.C., Mezêncio B., Soncin R., Filho C.A.C., Bottaro M., Hernandez A.J. (2018). CrossFit overview: Systematic review and meta-analysis. Sports Med. Open.

[11] Feito Y., Heinrich K.M., Butcher S.J., Poston W.S.C. (2018). High-intensity functional training (HIFT): Definition and research implications for improved fitness. Sports.

[12] Engel F.A., Ackermann A., Chtourou H., Sperlich B. (2018). High-intensity interval training performed by young athletes: A systematic review and meta-analysis. Front. Physiol..

[13] Gao Y., Wu M., Chen J., Feng Z., Wang X., Cao M. (2025). Effects of school-based concurrent training on body composition and cardiorespiratory fitness in obese children in a randomized controlled trial. Sci. Rep..

[14] Meier N., Schlie J., Schmidt A. (2023). Physiological effects of regular CrossFit^®^ training and the impact of the COVID-19 pandemic—A systematic review. Front. Physiol..

[15] Dominski F.H., Serafim T.T., Siqueira T.C., Andrade A. (2021). Psychological variables of CrossFit participants: A systematic review. Sport Sci. Health.

[16] Biddle S.J.H., Ciaccioni S., Thomas G., Vergeer I. (2019). Physical activity and mental health in children and adolescents: An updated review of reviews and an analysis of causality. Psychol. Sport Exerc..

[17] Lubans D., Richards J., Hillman C., Faulkner G., Beauchamp M., Nilsson M., Kelly P., Smith J., Raine L., Biddle S. (2016). Physical activity for cognitive and mental health in youth: A systematic review of mechanisms. Pediatrics.

[18] Liu J., Zhao Y., Cao M., Chen Z., Zhou H., Ke T., Chen Y., Tang C. (2026). Divergent effects of high-intensity functional training and moderate-intensity continuous training in adolescents with overweight/obesity: A randomized controlled trial on body composition, physical fitness, and psychological health. Front. Physiol..

[19] Eather N., Morgan P.J., Lubans D.R. (2016). Effects of exercise on mental health outcomes in adolescents: Findings from the CrossFit™ teens randomized controlled trial. Psychol. Sport Exerc..

[20] Faigenbaum A.D., Kraemer W.J., Blimkie C.J.R., Jeffreys I., Micheli L.J., Nitka M., Rowland T.W. (2009). Youth resistance training: Updated position statement paper from the National Strength and Conditioning Association. J. Strength Cond. Res..

[21] Lloyd R.S., Faigenbaum A.D., Stone M.H., Oliver J.L., Jeffreys I., Moody J.A., Brewer C., Pierce K.C., McCambridge T.M., Howard R. (2014). Position statement on youth resistance training: The 2014 International Consensus. Br. J. Sports Med..

[22] Vanaclocha-Amat P., Faigenbaum A.D., Molina-García J., Villa-González E. (2026). Strength and gamification in physical education: Effects of a gamified pediatric resistance training program on muscular fitness, enjoyment, and motivation in schoolchildren. Psychol. Sport Exerc..

[23] Kumar N.T.A., Oliver J.L., Lloyd R.S., Pedley J.S., Radnor J.M. (2021). The influence of growth, maturation and resistance training on muscle-tendon and neuromuscular adaptations: A narrative review. Sports.

[24] Sánchez-Pastor A., García-Sánchez C., Marquina-Nieto M., de la Rubia A. (2023). Influence of strength training variables on neuromuscular and morphological adaptations in prepubertal children: A systematic review. Int. J. Environ. Res. Public Health.

[25] Chan A.-W., Boutron I., Hopewell S., Moher D., Schulz K.F., Collins G.S., Tunn R., Aggarwal R., Berkwits M., Berlin J.A. (2025). SPIRIT 2025 statement: Updated guideline for protocols of randomised trials. BMJ.

[26] Hopewell S., Chan A.W., Collins G.S., Hróbjartsson A., Moher D., Schulz K.F., Tunn R., Aggarwal R., Berkwits M., Berlin J.A. (2025). CONSORT 2025 statement: Updated guideline for reporting randomised trials. BMJ.

[27] World Medical Association (2013). World Medical Association Declaration of Helsinki: Ethical Principles for Medical Research Involving Human Subjects. JAMA.

[28] International Council for Harmonisation of Technical Requirements for Pharmaceuticals for Human Use (ICH) (2016). Integrated Addendum to ICH E6(R1): Guideline for Good Clinical Practice E6(R2).

[29] Government of Spain (2007). Law 14/2007 of 3 July on Biomedical Research. Boletín Oficial del Estado.

[30] Government of Spain (2015). Royal Decree 1090/2015 of 4 December Regulating Clinical Trials with Medicinal Products, Ethics Committees for Research with Medicinal Products and the Spanish Clinical Studies Registry. Boletín Oficial del Estado.

[31] European Parliament, Council of the European Union (2016). Regulation (EU) 2016/679 of the European Parliament and of the Council of 27 April 2016 on the Protection of Natural Persons with Regard to the Processing of Personal Data and on the Free Movement of Such Data (General Data Protection Regulation). Off. J. Eur. Union.

[32] Government of Spain (2018). Organic Law 3/2018 of 5 December on Personal Data Protection and Guarantee of Digital Rights. Boletín Oficial del Estado.

[33] Faigenbaum A.D., McFarland J.E., Schwerdtman J.A., Ratamess N.A., Kang J., Hoffman J.R. (2006). Dynamic warm-up protocols, with and without a weighted vest, and fitness performance in high school female athletes. J. Athl. Train..

[34] Yelling M., Lamb K.L., Swaine I. (2002). Validity of a pictorial perceived exertion scale for effort estimation and effort production during stepping exercise in adolescent children. Eur. Phys. Educ. Rev..

[35] Léger L.A., Mercier D., Gadoury C., Lambert J. (1988). The multistage 20 metre shuttle run test for aerobic fitness. J. Sports Sci..

[36] Bosco C., Luhtanen P., Komi P.V. (1983). A simple method for measurement of mechanical power in jumping. Eur. J. Appl. Physiol. Occup. Physiol..

[37] Ruiz J.R., España-Romero V., Ortega F.B., Sjöström M., Castillo M.J., Gutierrez A. (2006). Hand span influences optimal grip span in male and female teenagers. J. Hand Surg. Am..

[38] Castro-Piñero J., Ortega F.B., Artero E.G., Girela-Rejón M.J., Mora J., Sjöström M., Ruiz J.R. (2010). Assessing muscular strength in youth: Usefulness of standing long jump as a general index of muscular fitness. J. Strength Cond. Res..

[39] Ortega F.B., Artero E.G., Ruiz J.R., España-Romero V., Jiménez-Pavón D., Vicente-Rodríguez G., Moreno L.A., Manios Y., Béghin L., Ottevaere C. (2011). Physical fitness levels among European adolescents: The HELENA study. Br. J. Sports Med..

[40] Tong T.K., Wu S., Nie J. (2014). Sport-specific endurance plank test for evaluation of global core muscle function. Phys. Ther. Sport.

[41] Polevoy G., Cazan F., Padulo J., Ardigò L.P. (2022). The influence of burpee on endurance and short-term memory of adolescents. Int. J. Environ. Res. Public Health.

[42] Maggio A.B.R., Vuistiner P., Crettenand A., Tabin R., Martin X.E., Beghetti M., Farpour-Lambert N.J., Deriaz O. (2017). Adapting the “Chester step test” to predict peak oxygen uptake in children. Swiss Med. Wkly..

[43] Ruiz J.R., Castro-Piñero J., España-Romero V., Artero E.G., Ortega F.B., Cuenca M.M., Jimenez-Pavon D., Chillon P., Girela-Rejon M.J., Mora J. (2011). Field-based fitness assessment in young people: The ALPHA health-related fitness test battery for children and adolescents. Br. J. Sports Med..

[44] Marfell-Jones M.J., Stewart A.D., de Ridder J.H. (2012). International Standards for Anthropometric Assessment.

[45] Spielberger C.D., Gorsuch R.L., Lushene R.E. (1973). Manual for the State-Trait Anxiety Inventory (STAI).

[46] Gualberto R., Seisdedos N., García C. (1982). STAIC: Cuestionario de Ansiedad Estado-Rasgo Para Niños.

[47] Trianes M.V., Blanca M.J., Fernández-Baena F.J., Escobar M., Maldonado E.F., Muñoz A.M. (2009). Evaluación del estrés infantil: Inventario Infantil de Estresores Cotidianos (IIEC). Psicothema.

[48] Rosenberg M. (1965). Society and the Adolescent Self-Image.

[49] Vázquez A.J., Jiménez R., Vázquez-Morejón R. (2004). Escala de autoestima de Rosenberg: Fiabilidad y validez en población clínica española. Apunt. Psicol..

[50] Riley A.W. (2004). Evidence that school-age children can self-report on their health. Ambul. Pediatr..

[51] Kang H. (2021). Sample size determination and power analysis using the G*Power software. J. Educ. Eval. Health Prof..

[52] Alves A., Marta C., Neiva H., Izquierdo M., Marques M. (2016). Concurrent training in prepubescent children: The effects of 8 weeks of strength and aerobic training on explosive strength and VO2max. J. Strength Cond. Res..

[53] Hedges L.V., Olkin I. (1985). Statistical Methods for Meta-Analysis.

