# Supplementary material for: High-Intensity Functional Concurrent Training for Physical Fitness, Body Composition, and Psychological Outcomes in Schoolchildren: Protocol for a Randomized Controlled Trial
Source: Sports (Basel). 2026 Jul 3;14(7):279. doi: 10.3390/sports14070279 (PMC13417676; doi:10.3390/sports14070279)
Supplement: Supplementary file 1 [file sports-14-00279-s001.zip › Figure S1.pdf]

SUPPLEMENTAL FILE

SPIRIT Schedule of Enrolment, Interventions, and Assessments

Figure S1. Schedule of enrolment, interventions, and assessments for the randomized controlled trial evaluating the effects of an 8-week HIFCT program in schoolchildren.

Procedures	Enrolment (-t1)	Allocation (0)	Pre (Week 0)	Intervention (Weeks 1–8)	Post (Week 8)
Eligibility screening	X				
Informed consent	X				
Medical history and PA questionnaire	X				
Randomization		X			
HIFCT intervention group		X		←——————→	
Control group		X		←——————→	
Anthropometry/body Composition			X		X
Muscular strength			X		X
Cardiorespiratory fitness			X		X
Psychological outcomes			X		X
(PCERT)			X	X	X

Supplementary Figure S1. Abbreviations: PCERT = Pictorial Children's Effort Rating, PA = Physical Activity, HIFCT = High Intensity Functional Concurrent Training
