# Supplementary material for: High-Intensity Functional Concurrent Training for Physical Fitness, Body Composition, and Psychological Outcomes in Schoolchildren: Protocol for a Randomized Controlled Trial
Source: Sports (Basel). 2026 Jul 3;14(7):279. doi: 10.3390/sports14070279 (PMC13417676; doi:10.3390/sports14070279)
Supplement: Supplementary file 1 [file sports-14-00279-s001.zip › Figure S2.pdf]

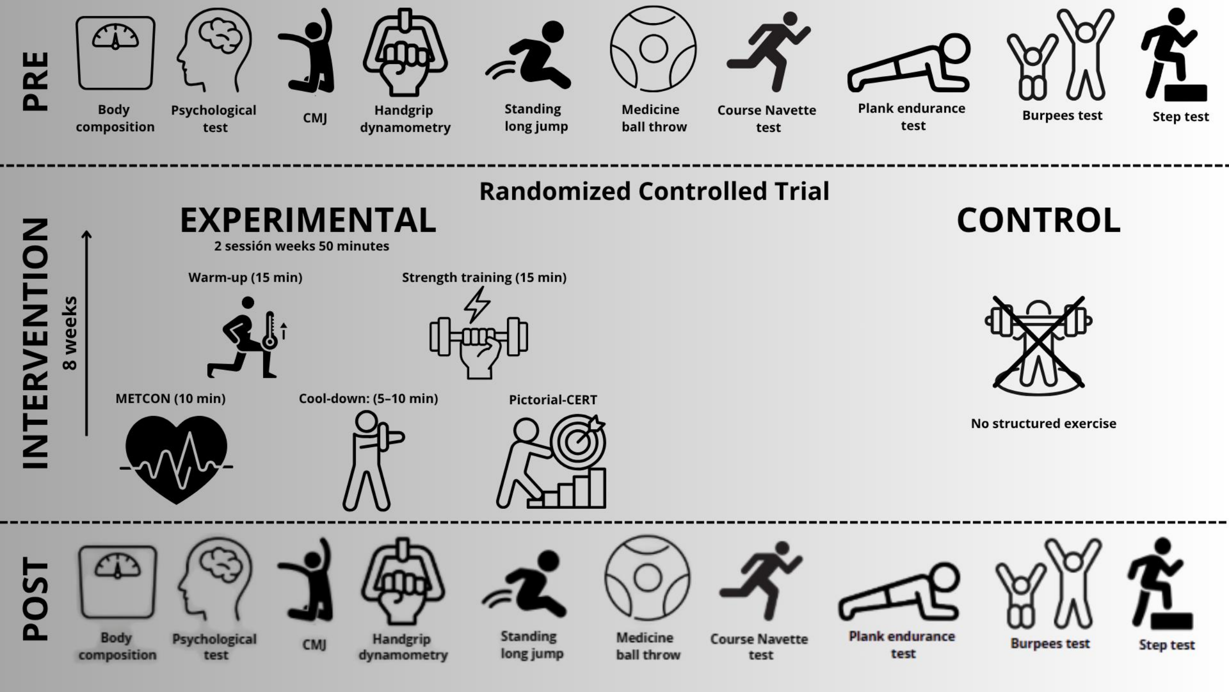
PRE
Body composition
Psychological test
CMJ
Handgrip dynamometry
Standing long jump
Medicine ball throw
Course Navette test
Plank endurance test
Burpees test
Step test
INTERVENTION
8 weeks
Randomized Controlled Trial
EXPERIMENTAL
2 sesión weeks 50 minutes
Warm-up (15 min)
Strength training (15 min)
METCON (10 min)
Cool-down: (5–10 min)
Pictorial-CERT
CONTROL
No structured exercise
POST
Body composition
Psychological test
CMJ
Handgrip dynamometry
Standing long jump
Medicine ball throw
Course Navette test
Plank endurance test
Burpees test
Step test
